# Application of Organic Amendments to a Coastal Saline Soil in North China: Effects on Soil Physical and Chemical Properties and Tree Growth

**DOI:** 10.1371/journal.pone.0089185

**Published:** 2014-02-18

**Authors:** Linlin Wang, Xiangyang Sun, Suyan Li, Tao Zhang, Wei Zhang, Penghui Zhai

**Affiliations:** 1 College of Forestry, Beijing Forestry University, Beijing, China; 2 Forestry Research Institute of Hulun Buir, Hulun Buir, Inner Mongolia, China; DOE Pacific Northwest National Laboratory, United States of America

## Abstract

The ability of the following four organic amendments to ameliorate saline soil in coastal northern China was investigated from April 2010 to October 2012 in a field experiment: green waste compost (GWC), sedge peat (SP), furfural residue (FR), and a mixture of GWC, SP and FR (1∶1∶1 by volume) (GSF). Compared to a non-amended control (CK), the amendments, which were applied at 4.5 kg organic matter m^−3^, dramatically promoted plant growth; improved soil structure; increased the cation exchange capacity (CEC), organic carbon, and available nutrients; and reduced the salt content, electrical conductivity (EC), and exchangeable sodium percentage (ESP). At the end of the experiment in soil amended with GSF, bulk density, EC, and ESP had decreased by 11, 87, and 71%, respectively, and total porosity and organic carbon had increased by 25 and 96% respectively, relative to the CK. The GSF treatment resulted in a significantly lower Na^+^+K^+^ content than the other treatments. CEC and the contents of available N, P, and K were significantly higher in the GSF-treated soil than in the CK and were the highest in all treatments. The FR treatment resulted in the lowest pH value and Ca^2+^ concentration, which decreased by 8% and 39%, respectively, relative to the CK. Overall, the results indicate that a combination of green waste compost, sedge peat and furfural residue (GSF treatment) has substantial potential for ameliorating saline soils in the coastal areas of northern China, and it works better than each amendment alone. Utilization of GWC and FR can be an alternative organic amendment to substitute the nonrenewable SP in saline soil amelioration.

## Introduction

Soil salinization is a major obstacle to the optimal utilization of land resources. Salt-affected soils are widely distributed throughout the world, and about 20% of the world’s cultivated land is salt-affected [1]. In the coastal regions of China, approximately 10^6^ hm^2^ of land is salt-affected because of sea water intrusion and the dry climate. The salt-affected soils in the coastal areas of northern China have several unique characteristics including a high water table (saline groundwater is 0.5–1.0 m below the soil surface), a high groundwater mineralization degree (7–10 g·L^−1^), serious secondary salinization, and a low nutrient content [2]. The present extent of salt-affected soils substantially restricts plant growth in these areas.

Sodium (Na^+^) is the predominant soluble cation in most saline soils, particularly in the coastal areas. Most plants are hypersensitive to saline environments [3]. The tissues of plants growing in saline media generally exhibit an accumulation of Na^+^ and Cl^-^ and/or the reduced uptake of mineral nutrients, especially Ca^2+^, K^+^, N, and P [4]. Previous studies have reported on the adverse effects of excessive amounts of salts on the physical and chemical properties of soils and on plant growth and yield [5], [6]. Clark et al. (2007) [7] demonstrated that salt-affected soils exhibit poor soil structure resulting from soil physical processes such as slaking, swelling, and dispersion of clay. Tejada and Gonzalez (2005) [8] showed that an increase in electrical conductivity (EC) adversely affects soil total porosity, bulk density, and structural stability. Salinity also affects soil chemical properties such as pH, cation exchange capacity (CEC), exchangeable sodium percentage (ESP), soil organic carbon, and available nutrients [1]. Salinity also alters the osmotic and matric potential of the soil solution, thereby adversely affecting soil microbial communities and their activity [9]. Moreover, the changes in the proportions of exchangeable ions in the soil solution have osmotic and ion-specific effects that can produce imbalances in plant nutrients, including deficiencies of several nutrients or excessive levels of Na^+^ [4]. Reclaiming these salt-affected soils will require new amelioration methods and improved management practices [10].

Leaching with water, chemical amendment, and phytoremediation are among the methods used to ameliorate saline soils [10]. The use of gypsum, calcite, calcium chloride, and other chemical agents that provide Ca, which tends to replace exchangeable Na, is effective for saline soil amelioration [11]. Another important practice is the application of organic matter conditioners, which can both ameliorate and increase the fertility of saline soils [12]. Salt-affected soils generally exhibit poor structural stability due to low organic matter content. Many researchers have suggested that the structural stability of soil can be improved by the addition of organic materials (e.g. green manures, farmyard and poultry manures, compost, and food processing wastes) [13], [14]. Barzegar et al. (1997) [15] found that the application of organic matter to saline soils can accelerate Na^+^ leaching, increase the percentage of water-stable aggregates, and decrease the ESP, EC, and soil salinity. Wichern et al. (2006) [16] reported that the addition of maize straw to a saline soil reduced the negative effects of salinity on the microbial community and mineralization.

In China, green waste is mostly produced by urban landscape maintenance and represents a significant portion of municipal solid waste [17]. In view of both environmental protection and economic development, composting is the best method to manage and utilize green waste. Compost has a unique ability to improve the chemical, physical, and biological characteristics of soils. It improves water retention and soil structure by increasing the stability of soil aggregates [18]. Green waste compost (GWC) is a biologically stable, humus-like substance that can be used for saline soil amelioration. Peat is a partly-decomposed plant material that slowly accumulates in pond and lake bottoms and swamp areas. Sedge peat (SP) and sphagnum peat moss are the most common types of peat used for soil remediation, and the former is a fine-textured and more decomposed type of peat. Sedge peat can hold water and nutrients ten to fifteen times their own weight when fully saturated, yet still can hold 40% air. They have high soil CEC and pH buffering capacity owing to their great soil specific surface and chelation capacity [18]. The utilization of sedge peat as a soil amendment in agriculture represents a potentially large market for peat.

However, the use of peat must be reduced, because it is considered as a nonrenewable resource [19]. Thus, an alternative to peat must be developed to achieve the function of peat amendment and meet consumer’s environmental concern. Furfural residue (FR) is the by-product of furfural production; furfural is mainly obtained from corn cobs by acid catalysis at high temperature. Among organic amendments, FR has the highest potential for low-cost, high-volume production [20]. Because the cellulose and lignin in corn cobs are relatively stable, FR is enriched in cellulose and lignin [21]. The advantage of organic amendments (GWC, SP, and FR) is that it is unlikely to contain microorganisms that are pathogenic to humans, which is an important concern of such soil amendment [18], [22].

Even though there are many studies dealing with organic amendments as degraded soil conditioners, very little is known about their effects on coastal saline soil. In view of the above, three organic materials (GWC, SP, and FR) and five treatments: no amendment, GWC alone, SP alone, FR alone and a mixture of GWC, SP, and FR (abbreviated as GSF) were applied in this study. The aim of the work was to evaluate the effects of different organic amendments on soil salinity and fertility of saline soil as well as plant growth in the coastal areas of northern China. The information obtained from this study will help provide guidance on selection of organic matters in ameliorating coastal saline soil while considering their environmental concerns.

## Materials and Methods

### Ethics Statement

All necessary permits were obtained for the described field studies. The study was approved by the Dagang Agriculture and Forestry Administration Bureau, Tianjin, China.

### Experimental Site and Properties of the Organic Amendments

The research was conducted from April 2010 to October 2012 at the Coastal Salt-tolerant Plant Science and Technology Park (38°46′ N, 117°13′ E, 1.3 m a.s.l.), Dagang, Tianjin, China. The study site has a semi-humid continental monsoon climate. The mean annual temperature is 12.3°C, the highest monthly temperature is in July (26°C), and the lowest monthly temperature is in January (−4°C). On average, there are 2618 h of sunshine per year. The frost-free period is 211 d, and the global radiation is 121.1 MJ·m^−2^. The annual precipitation and evaporation are 593.6 mm and 1979.4 mm, respectively, and the mean relative humidity is 65%. Rainfall from June to September accounts for 84% of the total annual precipitation. The general properties of soil samples (0–25 cm depth) taken from the study site are shown in Table 1.

**Table 1:** Properties of the saline soil before organic amendments were applied.

Property	Value
pH	7.75 (0.22)
Electrical conductivity (dS·m^−1^)	3.69 (0.17)
Clay (g·kg^−1^)^a^	96 (9)
Silt (g·kg^−1^) a	680 (22)
Sand (g·kg^−1^) a	224 (15)
Bulk density (mg·m^−3^)	1.54 (0.01)
Total porosity (%)	43.46 (0.18)
Total N (g·kg^−1^)	0.69 (0.03)
Total C (g·kg^−1^)	3.69 (0.13)
C/N ratio	5.35 (2.00)
Available N (mg·kg^−1^)	44.33 (1.17)
Available P (mg·kg^−1^)	5.38 (0.59)
Available K (mg·kg^−1^)	143.33 (12.02)
Na^+^ (g·kg^−1^)^b^	2.40 (0.03)
Ca^2+^ (g·kg^−1^)^b^	1.25 (0.04)
Mg^2+^ (g·kg^−1^)^b^	0.38 (0.01)
K^+^ (g·kg^−1^)^b^	0.06 (0.01)
Cl^−^ (g·kg^−1^)^b^	2.81 (0.02)
SO_4_^2−^ (g·kg^−1^)^b^	2.38 (0.26)
HCO_3_^−^ (g·kg^−1^)^b^	0.33 (0.01)
Total salt (g·kg^−1^)^c^	8.74 (0.30)
Cation exchange capacity (cmol·kg^−1^)	15.70 (0.05)
Exchangeable Na (cmol·kg^−1^)	2.5 (0.2)
Exchangeable sodium percentage (%)	15.8 (0.2)

Values are means of four samples and standard errors are presented in parentheses.

a Determined using the methods described by Bao (2005) [25].

b Soluble salt ions.

c Total salt = Na^+^+ K^+^+ Ca^2+^+ Mg^2+^+ Cl^−^+ SO_4_^2−^+ HCO_3_^−.^

The organic amendments applied in this study were GWC, SP, FR, and GSF. The general properties of the organic amendments are shown in Table 2. The green waste used to prepare GWC consisted of fallen leaves and branch cuttings collected during greening maintenance. The waste was reduced to about 1 cm particle size with a grinder. Before composting, the moisture content of the raw material was adjusted to 60%, and the C/N ratio was adjusted to between 25 and 30 to optimize microbial activity [23]. In this experiment, the GWC was used when the primary fermentation was completed [24]. In SP treatment, sedge peat was obtained from The Garden Waste Consumption Centre of Chaoyang District (Beijing, China). FR was supplied by the Kain Furfural Corporation (Beijing, China). In GSF treatment, the GWC, SP, and FR were mixed at a ratio of 1∶1∶1 by volume.

**Table 2:** Properties of the organic amendments applied to the saline soil.

	Organic amendment			
Property†	GWC	SP	FR	GSF
pH	8.88 (0.03)	4.93 (0.08)	2.54 (0.03)	7.20 (0.03)
Electrical conductivity(dS· m^−1^)	6.82 (0.04)	1.54 (0.02)	2.99 (0.03)	3.36 (0.06)
Organic carbon(g·kg^−1^)	184 (5)	286 (10)	477 (15)	312 (8)
Total N (g·kg^−1^)	17.5 (0.04)	10.5 (0.01)	8.8 (0.12)	12.6 (0.22)
C/N ratio	10.5 (2.3)	27.2 (12.0)	54.2 (1.8)	30.0 (0.4)
Total P (g·kg^−1^)	3.20 (0.01)	0.07(0.01)	0.47 (0.03)	1.38 (0.03)
Total K (g·kg^−1^)	6.20 (0.03)	1.33 (0.02)	1.71 (0.02)	3.28 (0.11)
Na^+^ (g·kg^−1^)	7.38 (0.07)	0.34 (0.01)	0.29 (0.02)	2.96 (0.07)
K^+^ (g·kg^−1^)	11.38 (0.07)	0.03 (0.01)	1.04 (0.01)	4.63 (0.09)
Ca^2+^ (g·kg^−1^)	1.10 (0.07)	1.50 (0.05)	0.65 (0.03)	1.06 (0.09)
Mg^2+^ (g·kg^−1^)	2.26 (0.05)	0.98 (0.04)	7.81 (0.07)	3.76 (1.90)
Cl^−^ (g·kg^−1^)^a^	20.2 (4.2)	1.1 (0.1)	2.5 (0.6)	8.7 (0.3)
SO_4_^2−^ (g·kg^−1^)^a^	13.90 (4.00)	5.90 (0.05)	35.90 (7.10)	19.00 (0.09)

Values are means of six samples and standard errors are presented in parentheses.

GWC, green waste compost; SP, sedge peat; FR, furfural residue; GSF, green waste compost plus sedge peat plus furfural residue (1∶1∶1 by volume).

† Based on a dry weight except for the moisture content; ^a^ Determined by titration method [25].

### Experiment

The experiment was conducted with five treatments : (1) a non-amended control (CK), (2) addition of 14.2 kg·m^−3^ of GWC, (3) addition of 9.1 kg·m^−3^ of SP, (4) addition of 5.5 kg·m^−3^ of FR, and (5) addition of 8.1 kg·m^−3^ of GSF. The quantities of amendments applied per m^3^ were selected so that the same mass of organic matter (4.5 kg·m^−3^) was applied for each amendment. All treatments were arranged in a randomized complete block design with four blocks (78 m×12 m). Each block was again divided into five plots (12 m×12 m) and hence there were 20 (4×5) unit plots. The distance between two adjacent blocks and plots were 6 m and 3 m, respectively. The five treatments were randomly assigned to each plot within the individual blocks with a separate randomization for each block. The amendments were added to nine planting sites in each plot. The nine sites were evenly distributed based on a planting spacing of 3 m×3 m. Each planting site was 1 m×1 m×1 m. On April 25, 2010, the amendments were manually mixed with the soil in each site, and one 2-year-old Pagoda tree (*Sophora japonica* Linn) was planted in each site. Seedlings with similar height, basal diameter, and growth potential were obtained from the Coastal Salt-tolerant Plant Science and Technology Park. Each planting site was irrigated with 5 liters of water every other week from April 26, 2010 to June 25, 2010. All plots and planting sites were managed in the same manner. We measured the height and basal diameter of each Pagoda tree on October 10, 2010, October 12, 2011, and October 14, 2012.

### Soil Sampling and Analyses

An auger was used to collect one soil core (0–25 cm depth, 5 cm diameter) from the amended zone of each of three randomly selected trees in each plot on October 11, 2010, October 13, 2011, and October 15, 2012. After drying, the soil samples were ground to pass through a 0.25-mm sieve and stored in sealed polyethylene bags at 4°C until chemical analysis.

Soil bulk density and total porosity were determined using the methods described by Bao (2005) [25]. pH and EC were determined with an MP521 pH/EC meter (Shanghai, China) for a slurry consisting of 1∶5 (W/V) soil/distilled water. Soil organic carbon content was determined by the potassium dichromate wet-combustion method. Total N was determined using the micro-Kjeldahl method. Total P was determined with a colorimetric method [25]. Total K was measured with flame photometric detectors. Available N was determined with a micro-diffusion technique after alkaline hydrolysis. Available P was extracted using 0.5 M NaHCO_3_ and was measured spectrometrically. Available K was measured by flame photometry after NH_4_OAc neutral extraction [26].

Soluble Na^+^ and K^+^ were determined with a flame photometer, and Ca^2+^ and Mg^2+^ were measured by titration with EDTA [27]. The CEC was determined with a 1 M ammonium chloride solution in ethanol/water (60∶40, v/v) at pH 8.2 [28]. Extracted Na was determined by flame photometry.

The ESP was determined by the formula,
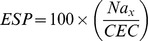
where Na_x_ is the exchangeable sodium (cmol·kg^−1^) and CEC is the cation exchange capacity (cmol·kg^−1^). Exchangeable sodium (Na_x_) was determined with 1 M ammonium acetate at pH 7 [29].

### Data Analysis

To test the differences among the treatments, randomized block split-plot analysis of variance (ANOVA) was used with soil amendment treatments as the fixed factor and experimental blocks as the random factor. The probability level used to determine significance was *p*<0.05. If treatment effects were significant, means were compared with an LSD test at *p*≤0.05. All statistical analyses were performed using SPSS software package (version 20.0) (IBM Corporation, Armonk, New York).

## Results

### Bulk Density, Total Porosity, EC, and ESP as Affected by Organic Amendments

**Figure 1:**
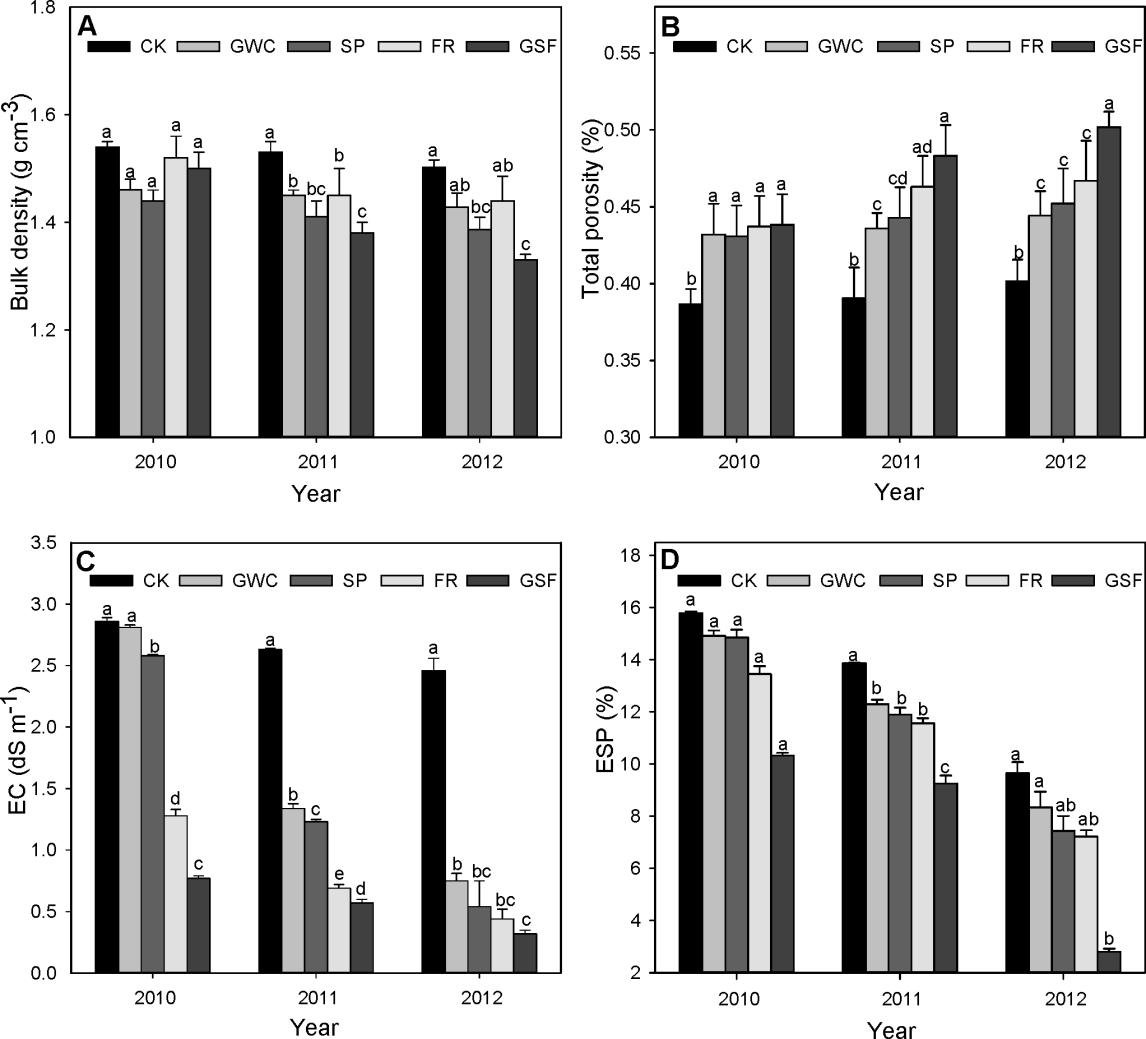
Effects of organic amendments on (A) bulk density, (B) total porosity, (C) electrical conductivity (EC), and (D) exchangeable sodium percentage (ESP) in saline soil. CK, non-amended control; GWC, green waste compost; SP, sedge peat; FR, furfural residue; GSF, green waste compost plus sedge peat plus furfural residue. Rates are indicated in the text but all amendments provided 4.5 kg of organic matter m^−3^. Treatments were applied in April 2010, and samples were collected in October 2010, October 2011, and October 2012. Values are means+SEMs. Means in the same year followed by the same letter are not significantly different at *p*≤0.05 according to LSD.

In October 2010, about 5.5 months after amendments were applied, there was no significant difference in soil bulk density among the treatments (Fig. 1A). In October 2011, bulk density was significantly lower in all amended plots than in the control (CK). In October 2012, bulk density was significantly lower in SP and GSF plots than in the control. In general, bulk densities were lowest in plots treated with GSF. All four amendments significantly increased the total soil porosity relative to the control (Fig. 1B). Total soil porosity was highest in GSF plots.

By the second year of the study, all four amendments had significantly reduced soil EC values relative to the control (Fig. 1C). EC values dropped more slowly in GWC and SP plots than in FR and GSF plots, but by 2012, the EC values had dropped to a lower level in all plots treated with amendments.

The initial ESP value in the CK plots was 15.8%, which exceeded the critical sodicity value (≈15%) mentioned by Richards (1954) [29]. ESP tended to drop in all plots including the CK plots over time (Fig. 1D). In 2011, ESP values were lower in all amended plots than in the CK plots and were lowest in the GSF plots. In 2012, ESP values were lowest in the GSF plots and did not significantly differ among the other plots.

### Soil pH, Organic Carbon, and CEC as Affected by Organic Amendments

**Figure 2:**
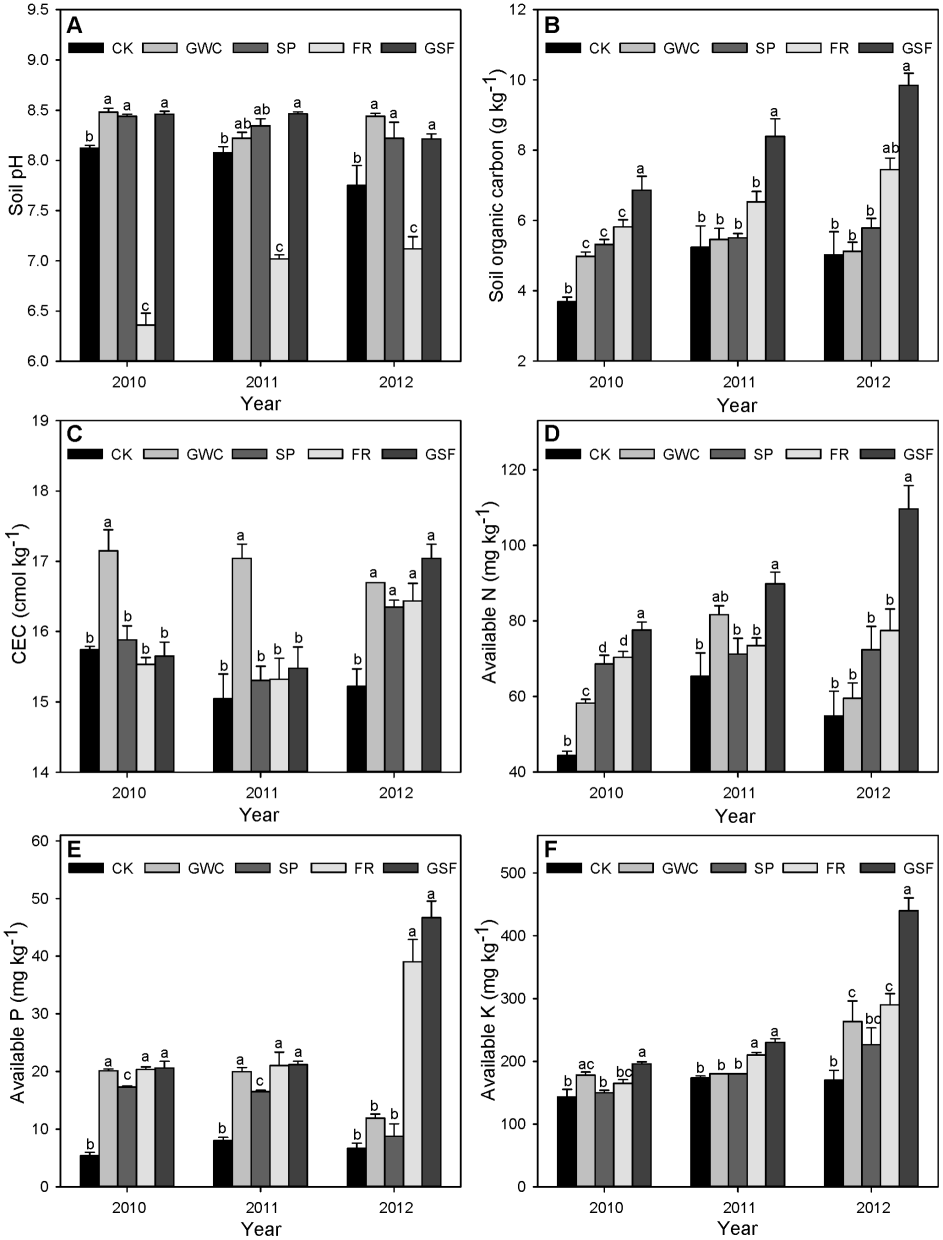
Effects of organic amendments on (A) soil pH, (B) soil organic carbon, (C) cation exchange capacity (CEC), (D) available N, (E) available P, and (F) available K in saline soil. CK, non-amended control; GWC, green waste compost; SP, sedge peat; FR, furfural residue; GSF, green waste compost plus sedge peat plus furfural residue. Rates are indicated in the text but all amendments provided 4.5 kg of organic matter m^−3^. Treatments were applied in April 2010, and samples were collected in October 2010, October 2011, and October 2012. Values are means+SEMs. Means in the same year followed by the same letter are not significantly different at *p*≤0.05 according to LSD.

In 2010 and 2012, FR decreased soil pH while the other three organic amendments increased pH relative to the control (Fig. 2A). The trends were similar in 2011 but were not always statistically significant. The effect of FR on pH tended to lessen from 2011 to 2012 and especially from 2010 to 2011. Soil pH did not significantly differ among GWC, SP, and GSF plots throughout the experiment.

In 2010, 5.5 months after the amendments were applied, soil organic carbon contents were significantly higher in all amended plots than in the CK plots and were highest in GSF plots (Fig. 2B). In 2011 and 2012, soil organic carbon was higher in GSF plots than in the other plots and did not significantly differ among the other plots, although soil organic carbon tended to also be high in FR plots.

In 2010 and 2011, soil CEC was higher in GWC plots than in the other plots and did not significantly differ among the other plots (Fig. 2C). In 2012, soil CEC was higher in all amended plots than in CK plots and did not significantly differ among the amended plots.

### Available Soil Nutrients as Affected by Organic Amendments

The content of available N was significantly higher in the GSF plots than in the other plots, except in 2011, when the level of available N was higher in GSF plots than in all other plots except GWC plots (Fig. 2D). The content of available P was increased by all amendments in 2010 and 2011 (Fig. 2E); in 2012, available P increased to very high levels in FR and GSF plots but did not differ from that in CK plots for the other two amendments. In 2010, the content of available K was greater in GWC and GSF plots than in CK plots but did not differ among SP, FR, and CK plots (Fig. 2F). In 2011, available K was highest in GSF and FR plots and did not differ among the other plots. In 2012, available K increased much more in GSF plots than in the other plots. The level of available K was also significantly higher in GWC and FR plots than in CK plots.

### Soluble Cations in the Soil as Affected by Organic Amendments

Table 3 shows the concentration of soluble cations in the soil on October 11, 2010 (about 5.5 months after amendments were applied) and at the end of the experiment on October 15, 2012 (about 29.5 months after treatments were applied). For all treatments including CK, the concentration of Na^+^+K^+^ in October 2012 was lower than that in October 2010 (*p*<0.05). The Na^+^+K^+^ concentration in October 2012 was highest in CK plots; intermediate in GWC, SP, and FR plots; and lowest in GSF plots. In addition, the proportion of soluble cations represented by Na^+^+K^+^ (Na^+^+K^+^/total) tended to decline in plots treated with organic amendments but tended to increase in CK plots. The Na^+^+K^+^/total value in October 2012 was lowest in the GSF plots. Like Na^+^+K^+^ concentrations, Ca^2+^ and Mg^2+^ concentrations tended to be lower in October 2012 than in October 2010; the drop in Ca^2+^ concentration was greatest in CK plots. The Ca^2+^ concentration in October 2012 was lowest in FR plots but did not statistically differ between the CK plots and those treated with organic amendments. Mg^2+^ concentrations did not significantly differ among treatments in October 2010 or in October 2012. Unlike the proportion of soluble cations represented by Na^+^+K^+^ (Na^+^+K^+^/total), the proportion of soluble cations represented by Ca^2+^ (Ca^2+^/total) tended to decrease in the CK plots and increase in the plots treated with organic amendments. In October 2012, the Ca^2+^/total value was lowest in CK plots; intermediate in SP, FR, and GSF plots; and highest in GWC plots (Table 3).

**Table 3:** Soluble cation contents of the soil in 2010 (about 5.5 months after amendments were applied) and in 2012 (about 29.5 months after amendments were applied).

	Na^+^+K^+^ (g·kg^−1^)		Ca^2+^ (g·kg^−1^)		Mg^2+^ (g·kg^−1^)		Na^+^+K^+^/Total^a^ (%)		Ca^2+^/Total^a^ (%)	
Treatment	2010	2012	2010	2012	2010	2012	2010	2012	2010	2012
CK	2.46(0.03)a	2.17(0.11)a	1.25(0.13)a	0.28(0.02)ab	0.38(0.05)a	0.08(0.02)a	0.60(0.01)e	0.86(0.03)a	0.31(0.02)a	0.11(0.01)c
GWC	1.66(0.03)b	0.38(0.04)b	1.12(0.04)a	0.45(0.01)a	0.36(0.03)a	0.09(0.01)a	0.53(0.01)d	0.41(0.02)b	0.36(0.03)a	0.51(0.01)a
SP	1.61(0.02)b	0.36(0.03)b	0.98(0.02)ab	0.41(0.05)ab	0.29(0.02)a	0.06(0.01)a	0.56(0.02)c	0.43(0.02)b	0.34(0.02)a	0.49(0.03)a
FR	1.48(0.03)b	0.37(0.02)b	0.43(0.01)b	0.17(0.03)b	0.30(0.01)a	0.06(0.02)a	0.67(0.02)a	0.62(0.02)c	0.19(0.01)b	0.28(0.01)b
GSF	0.89(0.02)c	0.17(0.01)c	0.82(0.03)ab	0.20(0.04)ab	0.32(0.02)a	0.05(0.01)a	0.44(0.02)b	0.40(0.03)b	0.40(0.01)a	0.47(0.02)a

CK, non-amended control; GWC, green waste compost; SP, sedge peat; FR, furfural residue; GSF, green waste compost plus sedge peat plus furfural residue (1∶1∶1 by volume).

Values are means (SE). Means in a column followed by the same letter are not significantly different at *p*≤0.05 according to LSD.

a Total = Na^+^+ K^+^+ Ca^2+^+ Mg^2+^.

### Tree Growth as Affected by Organic Amendments

**Figure 3:**
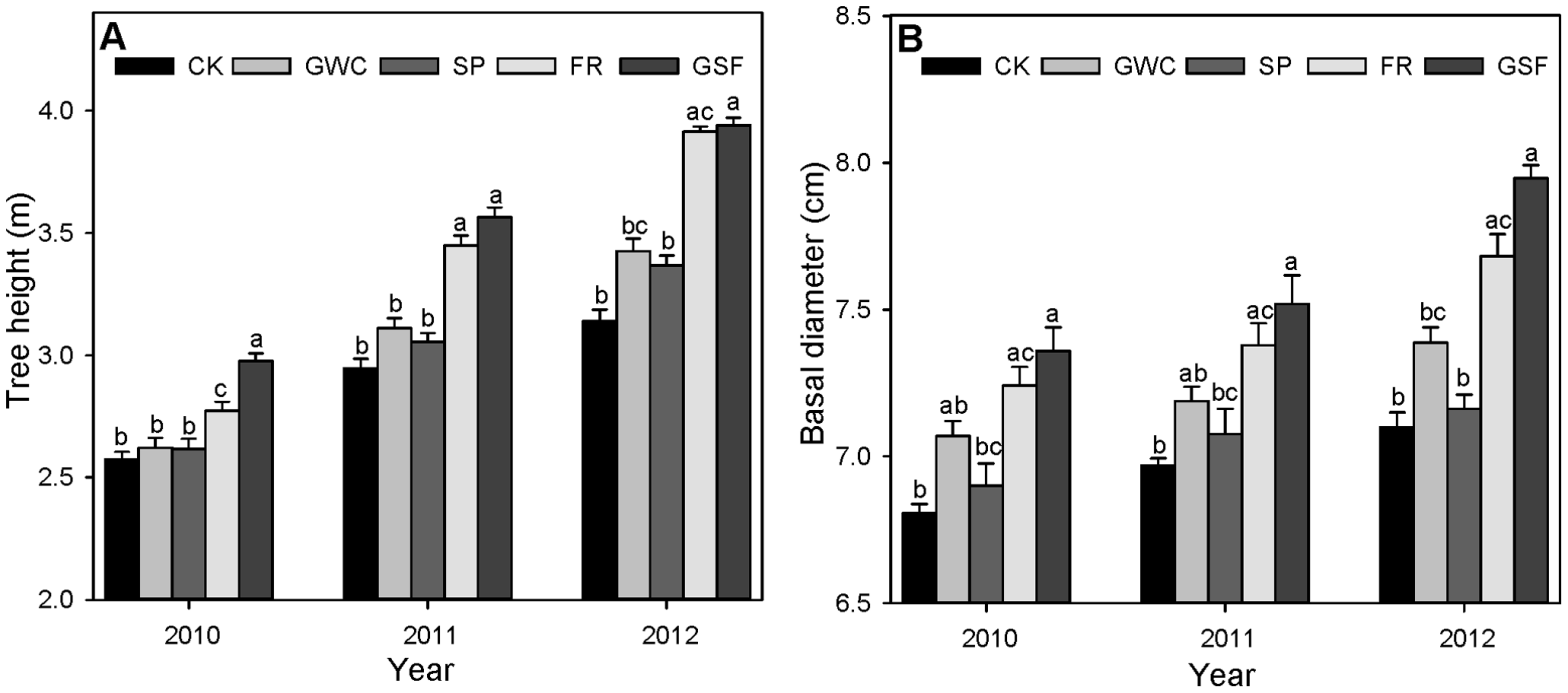
Effects of organic amendments on Pagoda tree (*Sophora japonica* Linn) (A) tree height, and (B) basal diameter in saline soil. CK, non-amended control; GWC, green waste compost; SP, sedge peat; FR, furfural residue; GSF, green waste compost plus sedge peat plus furfural residue. Rates are indicated in the text but all amendments provided 4.5 kg of organic matter m^−3^. Treatments were applied in April 2010, and data was determined in October 2010, October 2011, and October 2012. Values are means+SEMs. Means in the same year followed by the same letter are not significantly different at *p*≤0.05 according to LSD.

In our study, all amendments tended to enhance tree growth. In 2010, about 5.5 months after treatments were applied, tree height was greatest in GSF and FR plots but did not significantly differ among the other plots (Fig. 3A). The trends were similar in 2011 and 2012. Tree height did not statistically differ between GSF and FR treatments except in 2010. Basal diameter was higher in the GSF and FR plots than in CK plots, and did not differ between GWC, SP, and CK plots throughout the experiment (Fig. 3B). Like tree height, basal diameter did not statistically differ between GSF and FR treatments.

## Discussion

The GWC, SP, FR, and GSF amendments in the current study decreased soil bulk density and EC value and increased soil total porosity. This agrees with Oo et al. (2013) [14] and Tejada et al. (2006) [13], who found that organic amendments improve soil structure because they promote the flocculation of clay minerals, which is essential for the aggregation of soil particles. The GSF-treated soil had the lowest soil bulk density, the lowest EC, and the highest soil total porosity. These results have been explained by Oo et al. (2013) [14], who reported that combinations of organic amendments resulted in substantial flocculation and in the formation of a large number of soil aggregates. Improvements in soil structure can explain decreases in bulk density and increases in soil porosity [22], [30].

Soil EC values were negatively correlated with total porosity in the current study and in a previous study [14]. In addition, Kahlown et al. (2003) [6] and Barzegar et al. (1997) [15] found that the addition of organic matter to soil could increases leaching. This is probably related to the increased soil porosity resulting from the organic amendments [31]. Moreover, the four organic amendments used in our research, especially GSF and FR, contain calcium and magnesium (Table 2), which act as bases because they exist as oxides, hydroxides, and carbonates when applied to soil. As reported by Qadir et al. (2004) [32], an increase in the Ca^2+^ concentration in the soil solution results in the replacement of Na^+^ by Ca^2+^ at the cation exchange sites on the soil particles. Meanwhile, the greater the total porosity, the greater the leaching of the exchanged Na^+^ and the greater the subsequent reduction in soil salinity (as indicated by the EC value).

According to Mamedov et al. (2002) [33], ESP reflects soil aggregation. In the current study, the ESP value in the non-amended soil in 2010 was 15.8%, which indicates that swelling was the main mechanism determining its physical properties. In the amended soils, in contrast, ESP values were always <15%, suggesting that dispersion was probably the main mechanism determining their soil properties. Because ESP is inversely related to organic carbon content [13], it was not surprising that the plots with the lowest ESP values (the GSF plots) had the highest organic carbon contents.

Addition of GWC, SP, and GSF increased the pH of the saline soil, probably because these amendments had a high content of basic cations. Basic cations act in a similar manner as mineral lime, and they increase soil pH [34]. The FR plots had the lowest pH values probably because the FR was quite acidic (pH 2.54). The pH in the FR plots tended to increase over time, perhaps because the degradation of an organic amendment like FR that is rich in basic cations releases inorganic bases into the soil solution and thereby increases the soil pH [34].

Although the same mass of organic matter was added to the soils, the resulting increase in soil organic carbon differed depending on the amendment. This is consistent with Tejada et al. (2006) [30], who reported that the effect of organic amendments on soil organic carbon depended on the chemical nature of the amendments. On the one hand, the chemical nature of the organic amendment is likely to affect the rate at which it is decomposed by the microbial community [35]. On the other hand, the effects of the organic amendments on soil properties will affect plant growth and thus the input of plant residues into the soil [22], [36]. The organic carbon content of soil amended with GSF and FR increased gradually over time, and we assume that this increase reflected increased input from the trees (and perhaps reduced decomposition of that input).

CEC provides buffering against changes in pH, available nutrients, calcium levels, and soil structure [37]. The organic amendments used in our research led to an increase in soil CEC, especially in the GWC plots. This might have resulted from a high degree of organic matter oxidation in the GWC-amended soil, which could increase CEC values [38]. We also noted that, from 2010 to 2012, the CEC tended to increase in the SP, FR, and GSF plots. This may primarily be due to the long-lasting decomposition process of organic amendments [22].

Our findings showed that the organic amendments greatly increased the available N content. This agrees with previous reports that organic amendments increase available N [18], [39]. However, the effects of organic amendments on nutrient availability depend on their chemical composition and decomposition rates [7]. In the current study, GSF resulted in the highest level of available N, because the mixed organic amendments contain higher percentage of macronutrients and considerable amounts of micronutrients [40]. The C/N ratio was lower in the GWC amendment than in the other organic amendments (Table 2), and a low C/N ratio (less than about 20∶1) would result in intense microbial activity, and an increase in available N levels [41]. The available N content was relatively constant in the SP plots, this was probably because the highly-decomposed sedge peat decomposed slowly when added to soil [18]. The FR plots had the second highest level of available N at the end of the experiment, perhaps because the FR amendment had the highest organic matter content, and the organic matter in furfural is mineralized slowly over time [42].

The organic amendments significantly affected the levels of available P in the current study. Like available N content, available P content was highest in the GSF plots, perhaps because the mixed organic amendment has the ability to solubilize and mobilize P [43]. Moreover, P can be adsorbed by soil colloids, and the mixed organic amendment is able to bind large quantities of macronutrients and thereby to reduce their removal from soil by leaching [44]. P availability was also high in the FR plots, probably because the furfural residue was extremely acidic and thereby helped solubilize phosphate. In contrast, available P decreased in the GWC and SP plots probably because these amendments increased the pH.

The organic amendments also increased available K in the soil, which agrees with some researchers [45]–[47], who found relatively high levels of available K in soils treated with organic amendments. As was the case with available N and available P, available K was highest in the GSF plots and next highest in the FR plots. The difference in available K content among all amendments can be attributed to their chemical compositions. Thus, some K is released as a result of organic matter solubility and decomposition. Available K slightly increased in GWC and SP plots, perhaps because these amendments slowly release nutrients as they slowly decompose [18].

The organic amendments differed in their effects on the concentration of soluble cations. The application of organic amendments would probably increase the amount of Ca^2+^ derived from CaCO_3_ because of the formation of organic acids [48]. Furthermore, potassium and sodium ions on the soil colloids would be replaced by calcium ions and leached from the soil. The results revealed that, by the end of the experiment, the dominant ions changed from Na^+^ and K^+^ to Ca^2+^ in GWC, SP, and GSF plots. Mg^2+^ content also tended to decrease in all plots over time. Previous studies have reported that Ca^2+^ could improve soil structure by formed cationic bridges between clay particles and soil organic matter [49]. In addition, Ca^2+^ can inhibit clay dispersion and the associated disruption of aggregates by replacing Na^+^ and Mg^2+^ in clay and aggregates, thereby promoting aggregate stability [50]. These results indicate that GWC, SP, and GSF organic amendments could help reduce the salt content and otherwise improve saline soil. FR resulted in the lowest concentration of Ca^2+^ in 2010 and 2012, probably because the high SO_4_^2−^ concentration in the furfural residue promoted the precipitation of calcium sulphate [51].

The increased tree height and basal diameter observed in the GSF and FR treatments might be due to better physiological growth of plants. GSF and FR treatments increased the availability of macronutrients as well as micronutrients and improved soil physical properties of saline soil. Increased net primary productivity would have returned higher amounts of plant residues to the soil, perhaps acting as a carbon and nutrient source to maintain a higher microbial biomass and activity [36]. This, in conjunction with the positive effects of organic amendments on soil biota (e.g. increased biodiversity and microbial activity), would lead to continued nutrients release from organic amendments and soil amelioration [22], and help meet the amelioration goal of establishment of self-sustaining ecosystem on saline soil.

## Conclusions

The results of this study show that application of GWC, SP, FR, or GSF can improve the physical and chemical properties of saline soil and promote tree growth in the coastal areas of northern China. Application of GSF and FR substantially increased the organic carbon content of the soil and the availability of N, P, and K. The FR amendment effectively reduced the soil pH value. The GWC amendment increased the soil CEC and provided a long-term supply of nutrients. SP had a smaller effect on soil fertility than the other three amendments. Overall, the combination of GWC, SP and FR (the GSF treatment) provides better remediation results than each applied singly and has substantial potential for ameliorating coastal saline soils. The authors believe that SP could be fully or partially replaced by GWC and FR or their combinations in ameliorating coastal saline soil and that additional research should be conducted to optimize the mix proportion of GWC, SP, and FR. Considering the strong correlation between soil biological activities and soil quality [9], [16], further studies are also needed to assess the effects of organic amendments on soil microbial community and enzymatic activities.
